# Baseline Omega-3 Index Correlates with Aggressive and Attention Deficit Disorder Behaviours in Adult Prisoners

**DOI:** 10.1371/journal.pone.0120220

**Published:** 2015-03-20

**Authors:** Barbara J. Meyer, Mitchell K. Byrne, Carole Collier, Natalie Parletta, Donna Crawford, Pia C. Winberg, David Webster, Karen Chapman, Gayle Thomas, Jean Dally, Marijka Batterham, Ian Farquhar, Anne-Marie Martin, Luke Grant

**Affiliations:** 1 School of Medicine, University of Wollongong, Wollongong, New South Wales, Australia; 2 Illawarra Heath and Medical Research Institute, University of Wollongong, Wollongong, New South Wales, Australia; 3 School of Psychology, University of Wollongong, Wollongong, New South Wales, Australia; 4 South Coast Correctional Centre, Nowra, New South Wales, Australia; 5 School of Population Health, Sansom Institute for Health Research, Adelaide, University of South Australia, Adelaide, Australia; 6 Venus Shell Systems and Shoalhaven Marine & Freshwater Centre, University of Wollongong, Wollongong, New South Wales, Australia; 7 Statistical Consulting Service, University of Wollongong, Wollongong, New South Wales 2522, Australia; 8 Corrective Services New South Wales, Sydney, Australia; University of Medicine & Dentistry of NJ—New Jersey Medical School, UNITED STATES

## Abstract

**Background:**

There is emerging evidence that the supplementation of omega-3 contributes to a decrease in aggressive behaviour in prison populations. A challenge of such research is achieving statistical power against effect sizes which may be affected by the baseline omega-3 index. There are no published data on the blood omega-3 index with studies of this kind to assess the variability of the blood omega-3 index in conjunction with aggression and attention deficit assessments.

**Objective:**

To determine if the variance of the omega-3 index is correlated with aggressive and attention deficit behaviour in a prison population.

**Design:**

136 adult male prisoners were recruited from South Coast Correctional Centre (SCCC), NSW Australia. A 7 point categorisation was used to quantify levels of aggressive behaviour (4 weeks) from individual SCCC case notes, whereby higher scores correspond to increasingly aggressive behaviour. Study participants completed the Aggression Questionnaire (AQ) and the Brown’s Attention Deficit Disorder Scales (BADDS), provided a blood sample for erythrocyte fatty acid analysis using gas chromatography and the omega-3 index was calculated.

**Results:**

The baseline omega-3 index ranged from 2.3% to 10.3%, indicating that some participants already had substantial omega-3 intake, however a median of 4.7% indicated a lower overall omega-3 intake than the general Australian population. Assessment of aggressive and attention deficit behaviour shows that there were negative correlations between baseline omega-3 index and baseline aggression categorisation scores (r = −0.21, P = 0.016); total AQ score (r = −0.234, P = 0.011); Anger (r = -0.222 p = 0.016); Hostility AQ (r = −0.239, P = 0.009); indirect aggression (r = −0.188 p = 0.042); total BADDS (r = −0.263, p = 0.005); Activation (r = −0.224, p = 0.016); Attention (r = −0.192, p = 0.043); Effort (r = −0.253, p = 0.007); Affect (r = −0.330, p = 0.000) and Memory (r = −0.240, p = 0.010).

**Conclusions:**

There is a high variability in omega-3 status of a NSW prison population, and inmates with lower omega-3 index were more aggressive and had higher ADD scores.

## Introduction

In Australia, as in many Western countries, approximately half of all sentenced prisoners have a conviction of crimes of violence [1]. Reactive aggression, where injury and revenge are the primary goal, is the most common form of aggression among criminal offenders [2]. Reactive aggression is commonly associated with hostility [3], poorer metacognition [4] and impulsivity, and aggressive behaviour within Correctional Centres is of high concern and cost to both the individual offenders as well as Corrective Services. For example, threat perception among correctional officers is a significant contributor to overall well-being and influences work-related behaviour [5]. The populations in Australian and especially NSW correctional centres continue to rise, and the mental health needs of the prisoner population have been identified as being more considerable compared to the needs in the general population [6], as well as increased neurocognitive deficits such as attention deficit/hyperactivity disorder (AD/HD) [7] that are associated with poor impulse control. A multi-targeted approach at addressing this issue is a priority to provide for improved circumstances for the individuals within Correctional Centres, and potentially to contribute to prevention of ongoing mental health issues and re-offences.

Nutritious foods including long chain omega-3 polyunsaturated fatty acids (LC n-3 PUFA) are considered to be the building blocks of good mental health. In particular, it is well established that docosahexaenoic acid (DHA) plays an important role in neurological development [8]. Accordingly, low omega-3 status (in blood) is associated with increased mental illness [9,10]. A growing number of randomised controlled trials suggest that LC n-3 PUFA supplementation can ameliorate mental health issues such as hyperactivity, poor impulse control and depression across the lifespan [11]. At the later stage in life, LC n-3 PUFA are increasingly being linked to the delay or prevention of the onset of elderly mental health issues such as Alzheimer’s Disease and Dementia [12–14]. Post humous studies have indicated that mortalities associated with mental disorders in aged populations demonstrate significantly lower levels of omega-3 fatty acids, specifically DHA, in grey matter of the frontal lobe and hippocampus [15].

Australian’s (‘Western’) diet results in sub-optimal levels of omega-3 fatty acids [16,17], due to relatively reduced consumption of fish/seafood, the richest sources of LC n-3 PUFA [18]. Despite these low intakes of fish and hence LC n-3 PUFA, high intakes are associated with a lower likelihood of hostility in young adulthood [19]. The observed inverse relationship between apparent seafood consumption and homicide death when comparing 26 countries [20] and n-3 PUFA deficiencies may be associated with autonomic dysregulation associated with aggressive and hostile behaviour [21]. In support, two randomised controlled trials conducted in prison populations in Britain [22] and The Netherlands [23] supplemented prisoners with fish oil (containing LC n-3 PUFA) combined with multivitamins/minerals. They both reported 34–35% reductions in reprimands in the treatment groups compared with placebo, particularly for violent offences. However in the Dutch study [23] aggressive behaviour, with an average baseline score of 80 points, was reduced by 4.6 points in the active group whereas the placebo group reduced their scores by 1.8 points and was not significantly different (p = 0.09). However the trend in this study support the need for further research I this field, including consideration of the sources of variation that might contribute to a lack of statistical power in the case of a potentially false negative result.

One of the challenges in studies like these [22,23] is knowing the baseline condition of nutritional status and therefore the potential for an effect of intervention. For example it was not possible to know actual blood n-3 PUFA levels without blood samples, and therefore the potential for affecting the nutritional blood status and associating this effect with aggression or impulse control.

Further considerations are the tools applied to quantify aggression. There are numerous psychological tools that measure diverse aspects of behaviour; however the sensitivity and correlative relevance of these to blood status is poorly understood. The aim of this study was to determine the variance of the omega-3 levels of the blood in an extant prison population and determine if this baseline representation of the omega-3 levels is correlated with aggressive behaviour and psychological measures of trait aggression and poorer metacognitive processes in a prison population.

## Methods

### Ethics approval and recruitment of study participants

This study was approved by the Department of Corrective Services NSW, Australia Ethics committee (11/93185). Written consent was obtained from all study volunteers which was approved by the ethics committee. One hundred and thirty six adult, male study participants were recruited from the South Coast Correctional Centre (SCCC), Nowra, NSW, Australia for this study. Demographic information on the study participants was obtained by staff at the SCCC from the central database Offender Information Management System.

### Blood sampling

Baseline, non-fasted blood samples (serum and EDTA tubes) were collected by staff from Southern Pathology who attended the Correctional Centre on 3 consecutive days. Blood was taken into EDTA blood tubes which were subjected to low speed centrifugation and packed erythrocytes were frozen as aliquots at −80°C and later analysed for fatty acids.

### Erythrocyte fatty acid analysis

Erythrocyte samples were thawed and prepared for fatty acid analysis according to Swierk et al. [24]. Briefly, erythrocytes (400 μL) were resuspended in a TRIS buffer (10mM Bis Tris, 2mM EDTA Na_2_, pH 7.2) at room temperature for 30 mins. The samples were subjected to ultracentrifugation (30 mins, 49,000rpm, 4°C) in an Ultracentrifuge (Beckman, USA) to pellet the erythrocyte membranes. The supernatant was removed and discarded, and the erythrocyte membrane pellet was resuspended in 200μL distilled water. The direct transesterification procedure was then implemented according to Lepage and Roy [25]. Briefly, 2ml of methanol:toluene (4:1) (0.01% BHT) was added to 150μL of the erythrocyte sample, as well as 200μL of the internal standard, heneicosaenoic acid (0.2mg/mL, dissolved in toluene), and 200μL of acetyl chloride was added whilst vortexing. The tubes were tightly closed with teflon-lined caps, and heated at 100°C for 60 mins. The tubes were cooled in cold water, 5ml potassium carbonate (6%) was added, and tubes were centrifuged (10 mins, 3,000rpm, 4°C). The toluene (upper phase) of the sample was transferred into a gas chromatography (GC) vial, and stored at −20°C until analysis. Prepared erythrocytes samples were analysed by flame-ionisation gas chromatography (model GC-17A, Shimadzu) using a 50m x 0.25mm internal diameter capillary column. One microlitre of the sample was auto-injected into the column, and individual fatty acids were quantified using the Shimadzu analysis software (Class-VP 7.2.1 SP1, USA). Fatty acid peaks were identified by comparison with known fatty acid standards (Nu-chek and Sigma).

### Omega-3 Levels/Omega-3 Index

The omega-3 levels in the blood can be expressed as the Omega-3 Index, which was calculated as the sum of EPA and DHA expressed as percent of total erythrocyte fatty acids [26]. An omega-3 index of less than 4% is considered to be a risk factor for cardiovascular disease; a level between 4–8% is intermediate; and an omega-3 index greater than 8% is considered cardioprotective [26].

### Psychometric Assessments

• Behavioural Observations

Actual instances of hostile and aggressive behaviour are routinely recorded as prisoner incident reports, where an officer considers the behaviour substantial enough to warrant sanction. However, the intensity of such behaviour required to warrant sanction fails to capture the range and diversity of hostile and aggressive behaviours and thus results in a low incidence rate. Further, different jurisdictions have developed different metrics for the classification of behavioural disorder. Thus, for this study we developed a generic behavioural observation rating scale: the Inmate Behaviour Observation Scale (IBOS). The IBOS is a 7 point scale which classifies inmate behaviours across a four week period using data derived from individual SCCC case notes. Case notes are routinely recorded by custodial and non-custodial staff in the Offender Integrated Management System database to report significant observations and interactions with prisoners. A score of −1 is applied to all instances recorded in that week of pro-social behaviour, while a score of 0 is given if there were no behaviours of significance recorded. Thereafter, instances of hostile/aggressive behaviour are scored from 1 (non-compliant) through to 5 (physically aggressive), with each level of hostility/aggression operationally defined and illustrated by examples. Thus, higher scores are associated with greater levels of aggressive behaviour. The IBOS was significantly correlated with the Aggression Questionnaire (AQ) and the Brown’s Attention Deficit Disorder Scales (BADDS) adding to its construct validity. The IBOS rating criteria are contained in S1 Table.

• Trait Aggression

Trait aggression was measured using the Aggression Questionnaire (AQ: [27]). The AQ is among the most widely researched self-report trait based measures of aggression, anger and hostility [28–30]. Furthermore, the AQ has been found to be reasonably robust with respect to social desirability [31], an issue some have argued limits the reliability of self-report measures of aggression [32]. Additionally, the AQ has been subjected to broad cross-cultural validation and is applicable to non-Anglo-Saxon samples [28].

The AQ comprises 34 items, each associated with a 5-point Likert scale, ranging from 1 (‘extremely uncharacteristic of me’) through to 5 (‘extremely characteristic of me’). The AQ yields five subscale scores: Physical Aggression; Verbal Aggression; Anger; Hostility; and Indirect Aggression. The AQ also yields a composite total score.

• Metacognition

The most common metacognitive deficit among prisoners is Attention Deficit/Hyperactivity Disorder (AD/HD: [33]). In keeping with AD/HD theories that promote the importance of dysexecutive syndromes, and in particular, working memory capacity [34], and with the observation that improved attention is associated with increased behavioural control in children with AD/HD [35], this study chose to focus on the attentional deficits associated with an AD/HD diagnosis.

Attention-deficit was assessed using the Brown Attention-Deficit Disorder Scales (BADDS: [36]). The BADDS is a 40-item self-report scale that measures a range of symptoms associated with inattention, but does not assess either hyperactivity or impulsivity. Compared to other rating instruments, the BADDS is effective at predicting clinical diagnosis [37]. Items are rated on a scale of 0 (never) through to 3 (almost daily) and combine to provide five subscales relating to executive function impairment: Activation (organizing, prioritizing, and activating work); Focus/Attention (focussing, sustaining, and shifting attention to tasks); Effort (regulating alertness, sustaining effort, and processing speed); Emotion/Affect (managing frustration and modulating emotions); and Memory (utilizing working memory and accessing recall). BADDS scores can range from 0 to 120, with increasing scores indicating more impairment. The clinical cut off score in adults is a BADDS total score of 50, producing 4% false negatives and 6% false positives [38].

Both the AQ and the BADDS were administered on site at the SCCC to the study participants in small groups over a 3 day period. A proportion of study participants completed the questionnaires in their cells and returned them to SCCC staff the following day.

Age adjusted t-scores were calculated from both questionnaires from their respective manuals for the purpose of data analysis.

### Statistical analyses

Data are presented using descriptive statistics and correlations are reported using Spearman’s Rho with bootstrapped confidence intervals. Analyses were conducted using IBM SPSS (V21, IBM Corporation, Armonk NY). The P values for the correlations were adjusted for multiple comparisons using the method of Benjamini and Hochberg [39]. Adjustments were conducted using the R statistical package (Version 3.1.0, 2014 R Core Development Team [40]. Baseline proportions of ethnicity are compared to the Australian prison population using exact tests.

## Results

### Patient demographics

The men had a mean age of 33 years, generally had low levels of education and approximately half were Caucasian (Table 1). The majority of our study sample (75%) was representative of the Australian prison population but our sample had lower proportion of Australian Aboriginals and higher Asians, Hispanics and Polynesians.

**Table 1:** Demographic characteristics of the study participants.

	Study	Australian prisoners
	participants	As at 30 June 2013
	(n = 136)	(n = 30,775)
Age in years (mean (SD))	33 (11)	33.9 (median)
(range)	(18–80)	
Education (number (%))		Not reported
Primary School	6 (5.7)	
Lower High School	68 (65)	
Upper High School	23 (22)	
Tertiary	8 (7.6)	
Ethnicity (number (%))		
Arabic	11 (8)	539 (1.8)
Asian	12 (9)	1,600 (5.2)*
Australian Aboriginal	14 (10)	8,430 (27)*
Caucasian	74 (54)	18,490 (60)
Hispanic	5 (4)	113 (0.4)*
Polynesian	13 (10)	88 (0.3)*
Unknown	7 (5)	1,444 (4.7)

* Proportions significantly different between this sample and the Australian Prison population. Overall P value 0.000 using an exact test difference between column proportions assessed using post hoc z tests.

Values are mean and standard deviation (SD) for continuous variables or number (%) for categorical variables.

Despite the huge range on the IBOS, the scores of incidents across inmates were highly skewed with 69 inmates having a baseline score of zero resulting in a median score of zero (Table 2). The mean subscales of the scores (adjusted T-scores) of the AQ ranged from 53 to 58 with the overall total aggression mean of 55. The mean subscale scores (adjusted T-scores) of BADDS ranged from 59 to 62 with the overall total BADDS mean of 62.

**Table 2:** Measures of Aggressive and Attention Deficit Behaviours (adjusted T-scores) and the Omega-3 Index.

Measures of Aggressive Behaviour	Mean (SD)
7 point scale of aggressive behaviour (IBOS)	1.1 (2.7)
Median, (IQR)	0 (0, 1)
range (n = 136)	−3 to 15
Aggression Questionnaire (n = 117)	
Physical Aggression	58 (11)
Verbal Aggression	53 (10)
Anger	56 (12)
Hostility	57 (11)
Indirect Aggression	54 (11)
Total Aggression	55 (10)
BADDS (n = 112)	
Activation (n = 115)	60 (12)
Attention (n = 112)	62 (13)
Effort (n = 114)	59 (12)
Affect (n = 114)	61 (12)
Memory (n = 114)	62 (12)
Total BADDS (n = 112)	62 (14)

**Fig 1:**
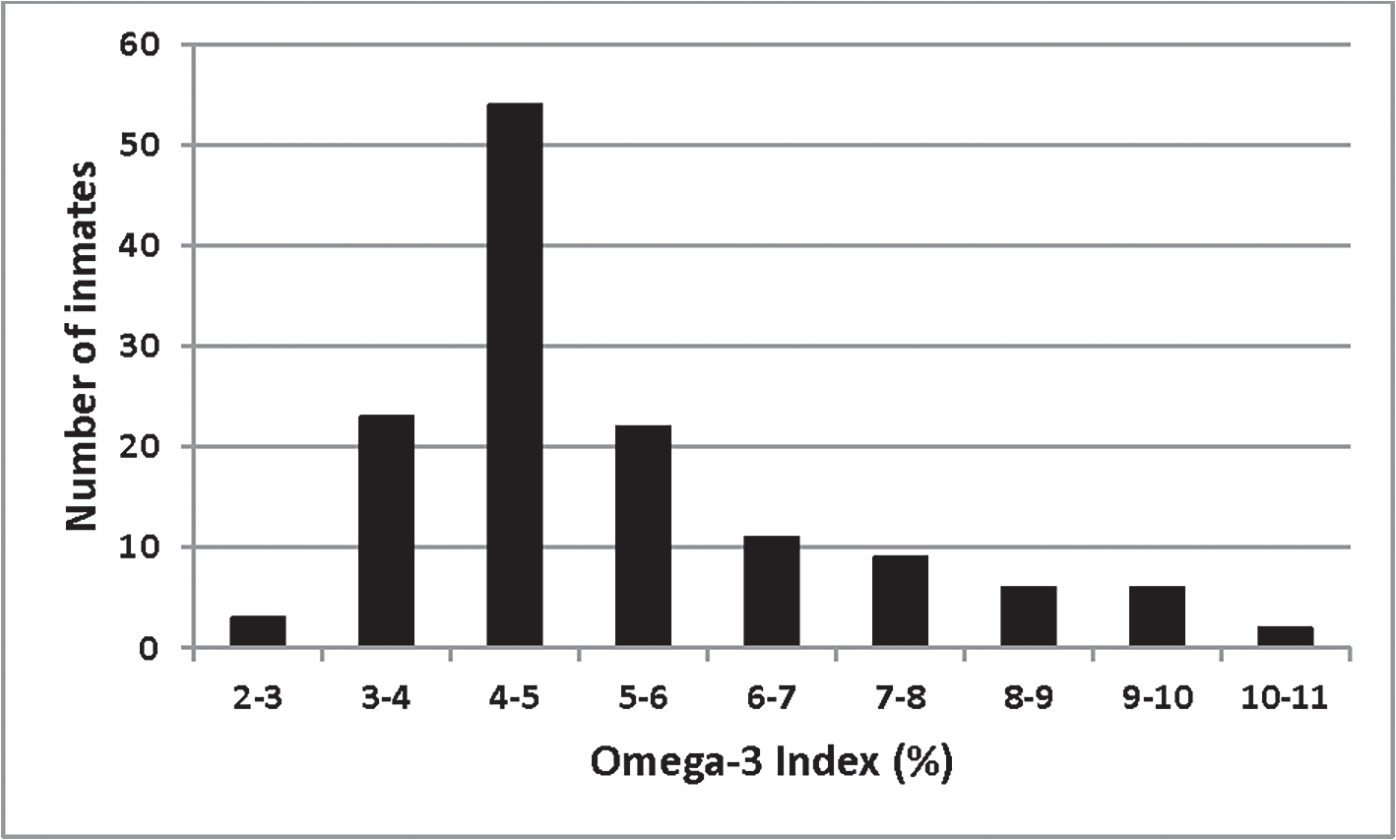
Distribution of the baseline Omega-3 Index. The Omega-3 Index was calculated as the sum of EPA and DHA expressed as percent of total erythrocyte fatty acids [26].

The median blood Omega-3 Index of participants was 4.7%, with a large range from very low at 2.3% to as high as 10.3% (Fig. 1). Twenty-six inmates (19%) had an omega-3 index less than 4% and 14 inmates (10%) had an omega-3 index greater than 8%. Thirty-four of the inmates (25%) had a baseline omega-3 index of 6% or greater.

**Fig 2:**
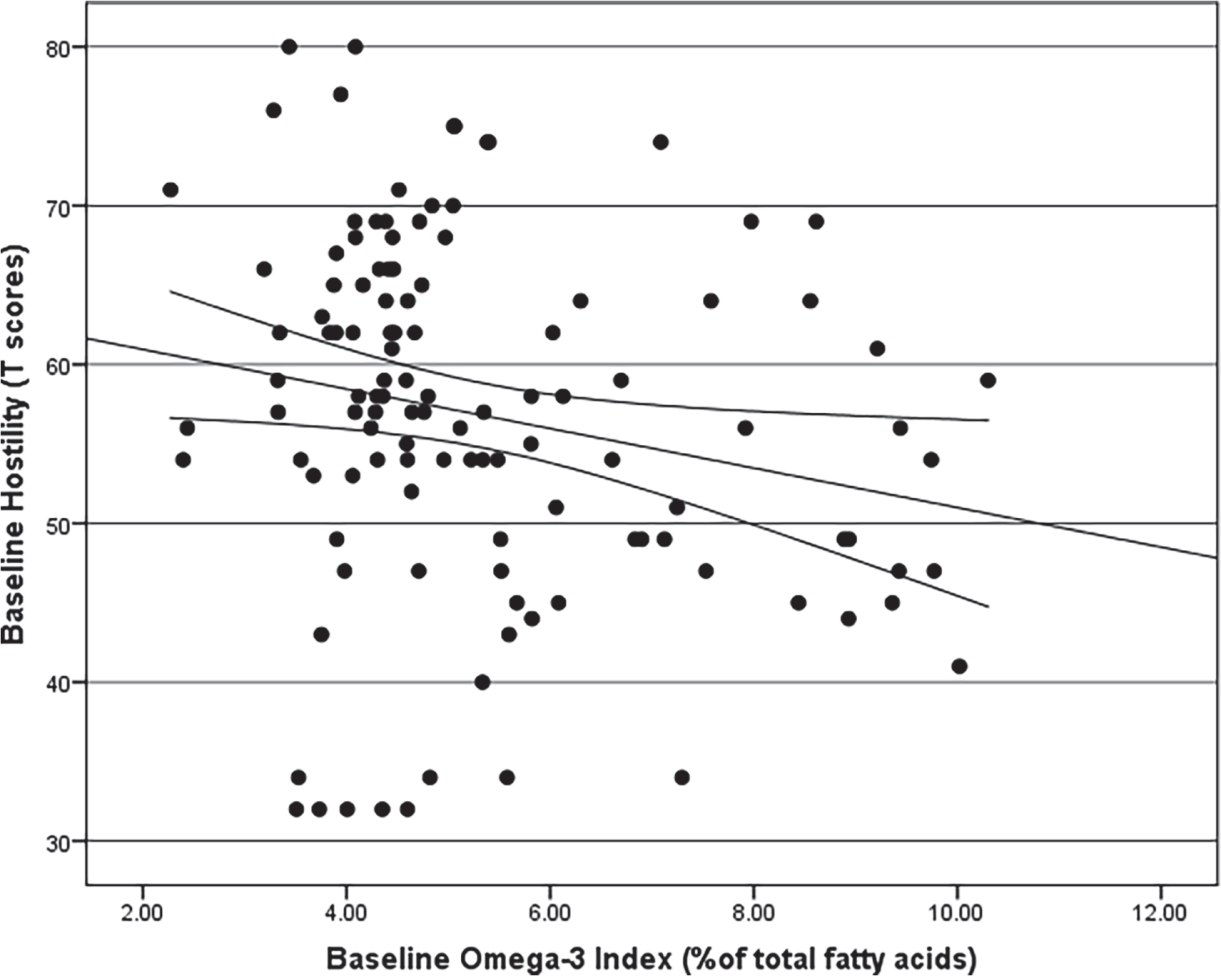
Spearman’s Rho Correlation with bootstrapped confidence intervals between Hostility and Omega-3 Index (r = -0.239, adjusted p = 0.023).

**Fig 3:**
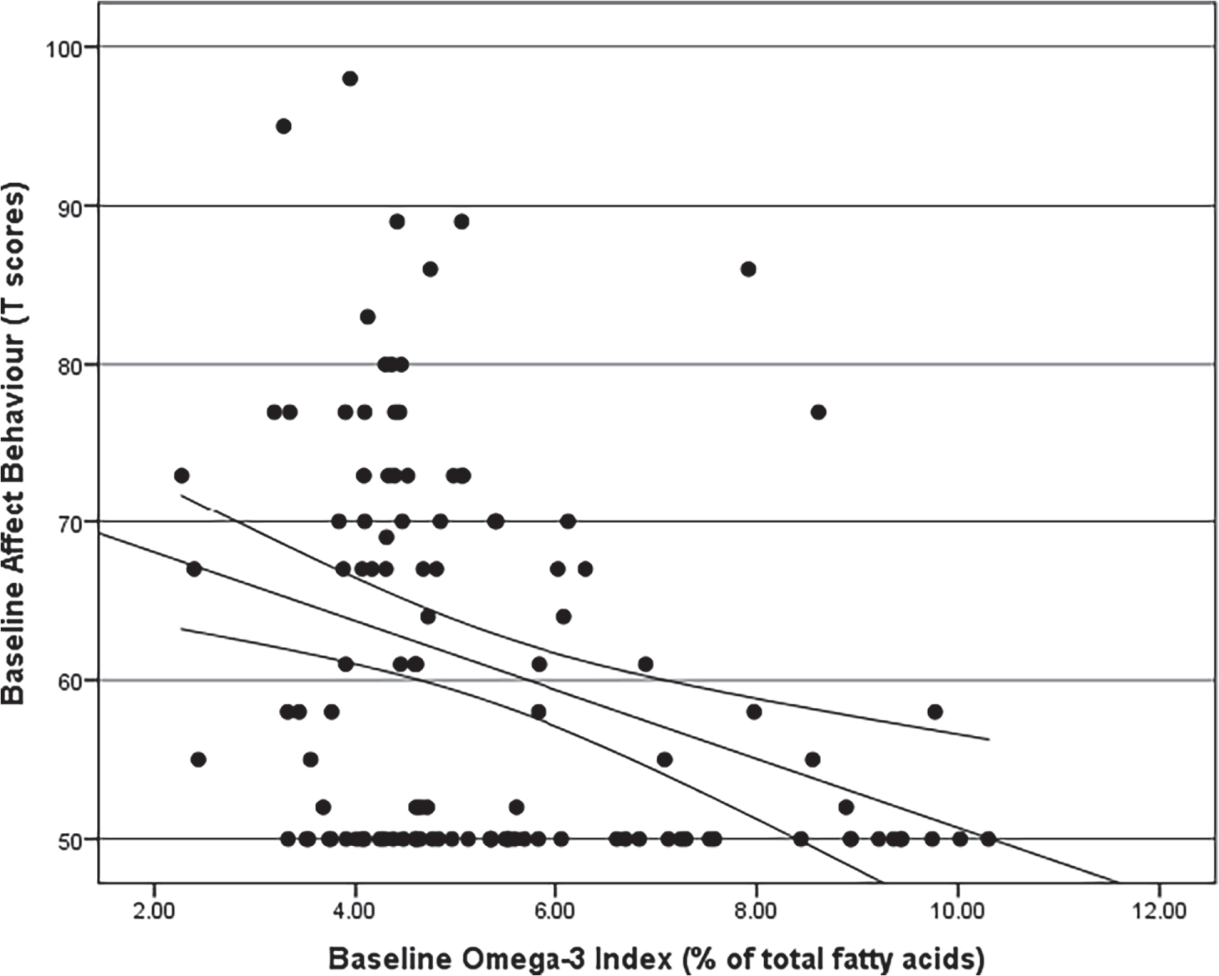
Spearman’s Rho Correlation with bootstrapped confidence intervals between Affect Behaviour and Omega-3 Index (r = -0.330, adjusted p = 0.000).

**Fig 4:**
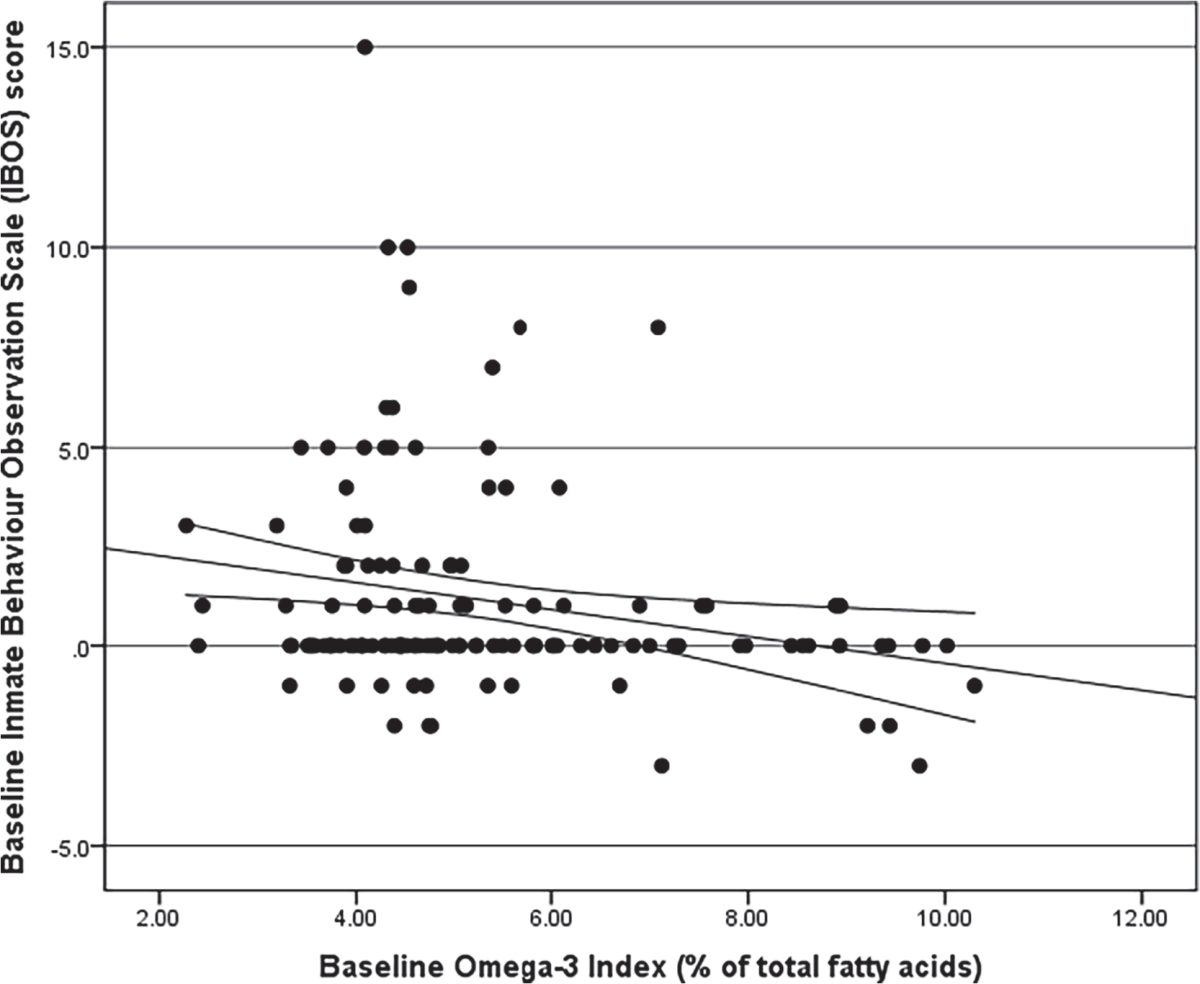
Spearman’s Rho Correlation with bootstrapped confidence intervals between the Inmate Observation Scale (IBOS) and Omega-3 Index (r = -0.207, adjusted p = 0.023).

Most measures of aggressive and attention deficit disorder behaviours were negatively correlated with the omega-3 index (Table 3), while 4 measures out of 11 (physical, verbal and indirect aggression as well as attention) did not significantly correlate with the omega-3 index, whilst all other measures of aggressive and attention deficit disorder behaviours were negatively correlated with the omega-3 index. Therefore, the lower the omega-3 index, the higher the score of aggressive and attention deficit disorder behaviours for the most part; the strongest negative correlations being between Hostility and Omega-3 index (Fig. 2), and Affect and Omega-3 Index (Fig. 3). The IBOS was also negatively correlated with and the Omega-3 Index (Fig. 4).

**Table 3:** Correlations between aggressive and attention deficit disorder behaviours and omega-3 Index.

Behaviour	Correlation co-efficient (95% CI)	P value	Adjusted P value
7 point scale of aggressive behaviour (IBOS) (n = 134)	−0.207 (−0.361, −0.048)	0.016	0.023
Total Aggression (n = 117)	−0.234 (−0.397, −0.051)	0.011	0.023
Physical Aggression (n = 117)	−0.174 (−0.352, 0.009)	0.060	0.065
Verbal Aggression (n = 117)	−0.159 (−0.315, 0.025)	0.087	0.087
Anger (n = 117)	−0.222 (−0.388, −0.047)	0.016	0.023
Hostility (n = 117)	−0.239 (−0.406, −0.054)	0.009	0.023
Indirect Aggression (n = 117)	−0.188 (−0.358, −0.015)	0.042	0.051
Total BADDS (n = 112)	−0.263 (−0.433, −0.080)	0.005	0.023
Activation (n = 114)	−0.236 (−0.399, −0.017)	0.016	0.023
Attention (n = 112)	−0.192 (−0.369, −0.001)	0.043	0.051
Effort (n = 114)	−0.253 (−0.425, −0.063)	0.007	0.023
Affect (n = 114)	−0.330 (−0.483, −0.160)	<0.001	<0.001
Memory (n = 114)	−0.240 (−0.417, −0.054)	0.010	0.023

P value adjusted for multiple comparisons using the method of Benjamini and Hochberg.

Bootstrapped confidence intervals are presented.

## Discussion

To our knowledge, our study is the first study to have taken blood samples and show that erythrocyte levels of omega-3 index negatively correlate with aggressive and attention deficit behaviours in prison inmates. There was a wide range of omega-3 index from as little as 2.3% to as high as 10.3%. The variance of omega-3 index in inmates is unlikely to be explained by the food supplied in the prison and time since incarceration. In the same correctional centre facility, inmates are generally provided similar food and given that the Australian population on average only consumes 26g of fish/seafood per day [18], this amount is not high enough to explain the high levels of omega-3 index. The high levels of the omega-3 index can be explained by the consumption of canned tuna and/or fish oil capsules which inmates can purchase from their available funds as a source of protein. The median omega-3 index of 4.7%, albeit slightly lower, is comparable to healthy Australian levels which have been reported at 5–6% [41]. However, these Australian levels are much lower than omega-3 index of countries like Japan (8.5%) and Korea (11%), where traditional diets contain large quantities of fish and seafood: the richest source of LC n-3 PUFA [18].

Previous studies [22, 23] showed that supplementation with multivitamins and minerals and LC n-3 PUFA resulted in approximately 30–35% reduction in the number and severity of reprimands in a prison population. However, these studies did not take blood samples to assess omega-3 status of the study participants. The LC n-3 PUFA are extremely important not only for heart health but also for cognition and mental health. Our recent review [8] describes in detail the mechanisms by which LC n-3 PUFA, in particular DHA exert their effect.

The main mechanism by which LC n-3 PUFA can exert its effects on aggressive behaviour is via its ability to increase neurotransmission [8]. For example, when the neurotransmitter dopamine binds to its receptor, protein kinase A activity increases, which results in phosphorylation of ion channels, opening them up and enabling the signal to be transmitted. When DHA is low, more dopamine is needed to have effective neurotransmission. Therefore for effective neurotransmission, humans need DHA in the cell membranes of their brains [8].

Aggressive and impulsive behaviours may be explained by suboptimal regulation of the limbic system in the frontal cortex, and LC n-3 PUFA insufficiency may reduce serotonergic function in the frontal cortex, explaining this relationship [21]. Research has linked lower n-3 PUFA concentrations in plasma with greater likelihood of aggressive and disruptive behaviours, with evidence emerging nearly 30 years ago that violent and impulsive offenders had lower plasma concentrations of DHA, compared with non-impulsive offenders and healthy controls [42]. Subsequent research has shown that boys aged 6 to 12 years with lower n-3 PUFA plasma concentrations exhibited greater behaviour problems [43], while levels of anger and anxiety among substance abusers were significantly reduced by n-3 PUFA supplementation, confirmed through pre/post supplementation n-3 PUFA blood plasma levels [9].

LC n-3 PUFA appear relevant to a broad range of neuropsychiatric disorders [44], including depression [45]. Depression is often co-morbid with aggression and hostility [46], and the improvement in overall affect as a result of adequate LC n-3 PUFA ingestion may also explain improvements in aggressive behaviour. The observation in our study of the strongest relationship being between Hostility (on the AQ) and Affect (on the BADDS) and Omega-3 Index would lend some support to this hypothesis.

The correlations that we have shown, for the first time in a prison sample, between the omega-3 index and BADDS subscales, including total BADDS, Activation, Attention, Effort, Affect, and Memory are supported by previous work suggesting links between n-3 PUFA and ADD/ADHD symptoms [47, 48]. Furthermore, previous research with children who had ADHD symptoms showed that improved objective assessments of cognition/attention were mediated by parent-reported improvements of not only attention, but also hyperactivity and impulse control [49]. This, plus the high comorbidity of ADD/ADHD with other disorders including conduct disorder suggests that executive functions such as attention are central to the cluster of symptoms associated with behavioural disorders that may also manifest as aggressive behaviour. These higher order functions are governed by the brain’s frontal lobes, which in previous work have been shown to respond to DHA supplementation during a sustained attention task [50]. We have discussed in another paper correlations between the AQ and the BADDS [33], further supportive of this supposition.

The strengths of this study are the blood analysis of the omega-3 status by measuring the Omega-3 Index and showing negative correlations with aggressive and attention deficit disorder behaviours, however these correlations show association but do not show cause and effect.

In summary, we have shown that there is high variability in the levels of omega-3 index in inmates in a NSW Correctional Centre, with the majority having levels below 6% of total erythrocyte fatty acids. The omega-3 index correlates negatively with levels of aggressive behaviour, especially hostility and also with attention deficit disorder behaviour especially affect. The significance of this link between diet and anti-social behaviour implies potential benefits for the correctional system and community safety at a population level. Supplementation with omega-3 and/or multi-vitamins and minerals is low cost and easy to administer compared to other forms of treatment for anti-social behaviour, and with further evidence from intervention trials could be a simple and effective contribution to treatment programs and the mental health status of offenders. Given these correlations and potential benefits, a multi-centre randomised controlled trial assessing the individual effects of omega-3 and multi-vitamins and minerals is warranted.

## Supporting Information

S1 Fig Leverage versus standardised residual plot of IBOS and baseline omega-3 levels. (TIFF)

S1 Table The Inmate Behavioural Observation Scale (IBOS) – criteria for behaviour rating. (PDF)
