# Correction: Baseline Omega-3 Index Correlates with Aggressive and Attention Deficit Disorder Behaviours in Adult Prisoners

**DOI:** 10.1371/journal.pone.0129984

**Published:** 2015-06-03

**Authors:** Barbara J. Meyer, Mitchell K. Byrne, Carole Collier, Natalie Parletta, Donna Crawford, Pia C. Winberg, David Webster, Karen Chapman, Gayle Thomas, Jean Dally, Marijka Batterham, Ian Farquhar, Anne-Marie Martin, Luke Grant

The following information is missing from the Funding section: Natalie Parletta (formerly Natalie Sinn) was supported by NHMRC Program Grant funding (# 320860 and 631947).
