# Correction: Baseline Omega-3 Index Correlates with Aggressive and Attention Deficit Disorder Behaviours in Adult Prisoners

**DOI:** 10.1371/journal.pone.0197231

**Published:** 2018-05-07

**Authors:** Barbara J. Meyer, Mitchell K. Byrne, Carole Collier, Natalie Parletta, Donna Crawford, Pia C. Winberg, David Webster, Karen Chapman, Gayle Thomas, Jean Dally, Marijka Batterham, Ian Farquhar, Anne-Marie Martin, Luke Grant

Information is missing from the first sentence of the “Ethics approval and recruitment of study participants” heading of the Methods section. The correct sentence is: This study was approved by the Department of Corrective Services NSW, Australia Ethics committee (11/93185) and the University of Wollongong Human Research Ethics Committee (NSA13/004).
