# Supplementary figures and images for: Baseline Omega-3 Index Correlates with Aggressive and Attention Deficit Disorder Behaviours in Adult Prisoners
Source: PLoS One. 2015 Mar 20;10(3):e0120220. doi: 10.1371/journal.pone.0120220 (PMC4368577; doi:10.1371/journal.pone.0120220)

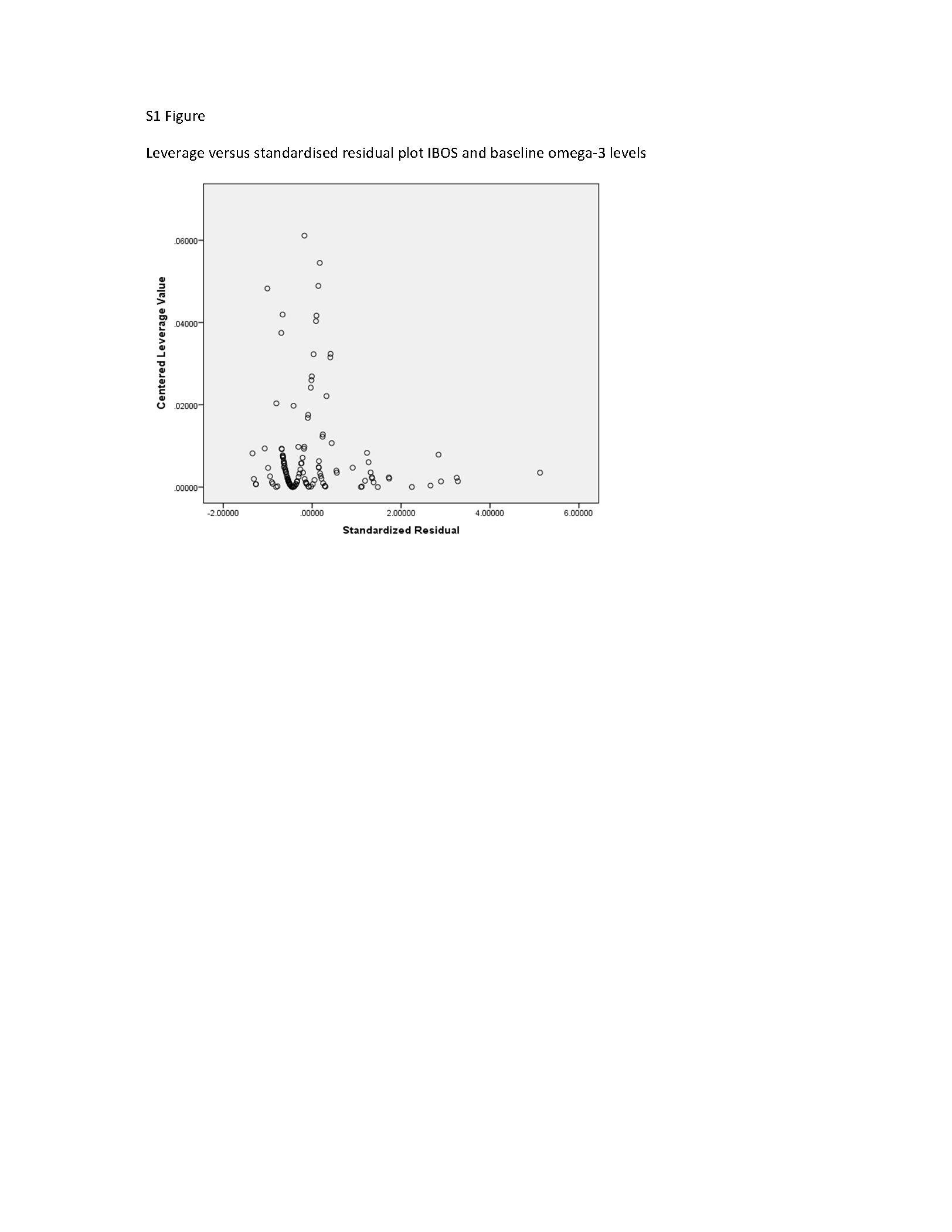

Supplement: S1 Fig — (TIFF) [file pone.0120220.s001.tiff]
