# Supplementary material for: Baseline Omega-3 Index Correlates with Aggressive and Attention Deficit Disorder Behaviours in Adult Prisoners
Source: PLoS One. 2015 Mar 20;10(3):e0120220. doi: 10.1371/journal.pone.0120220 (PMC4368577; doi:10.1371/journal.pone.0120220)
Supplement: S1 Table — (PDF) [file pone.0120220.s002.pdf]

S1 Table Inmate Behavioural Observation Scale (IBOS) - Criteria for Behaviour Rating

-1	0	1	2	3	4	5
Positive Behaviour: Any and all instances of independent positive behaviour by the inmate. It does not include vague statements like "appeared in a good mood" but actual instances of pro-social behaviour.	No events to record /relevant	Non Compliant Behaviour: there should be an absence of overt aggressiveness, either verbally or physically, or any apparent premeditation for aggression. Minor rule breaking or obstructiveness is rated	Contained Aggression: absence of any directed or indirect aggressiveness however negative attitudes expressed	Indirect Aggression: No person is directly identified as a target, although groups of people may be mentioned. Excludes possession of a weapon as there is an assumption of a known target.	Directed Aggression (non physical): Any situation where an identified target is involved but where no physical contact ensues.	Physical Aggression: Any instance where aggression results in contact or negative physical events for another
EXAMPLES EXAMPLES EXAMPLES						
Helping an officer without being asked and when not part of the inmates duties;<br><br>Assisting or helping another inmate (excludes victims of aggression);	Record this on the check sheet if no event occurs that day/ week	Smoking in non-smoking areas;<br><br>Failing to attend muster;<br><br>Failure to engage with medical or allied health treatment;<br><br>Demanding or excessive requests/threats of, or actual, self harm;<br><br>Requiring multiple prompts to follow a reasonable direction by an officer;<br><br>Possession of a contraband (e.g. mobile phone, drugs)<br><br>Failure to provide urine sample (not coded 4 unless case noted as refusal)	Saying "I don't give a fuck" when advised of consequences or asked to do something;<br><br>Repeatedly knocking up without aggression;<br><br>Disrupting the activities of others;<br><br>Being argumentative with officers	Known defiance to previous direction;<br><br>Destroying or damaging prison property;<br><br>Punching, kicking or slamming cell door;<br><br>Repeatedly knocking up with aggression;<br><br>Messing up cell or work station;<br><br>Making derogatory comments about staff in general	Blatantly defying staff after repeated current direction;<br><br>Verbally abusing a person who is present or identified by name;<br><br>Intimidation;<br><br>Threats to others;<br><br>Yelling/raising voice;<br><br>Possession of a weapon<br><br>Case noted to refuse to supply urine sample	Assaulting staff or other inmates;<br><br>Spitting on staff or other inmates;<br><br>Throwing things at staff or other inmates;<br><br>Brandishing a weapon at staff or other inmates

Week Beginning:_____________(date)   Participant_______________(code#)

Place a tick (✓) in each column for every separate and discrete occurrence of the behavioural category. Refer to the Behavioural Category Definitions. Use a separate sheet for each participant and for each weekly period.

Behavioural Category	-1	0	1	2	3	4	5
Monday							
Tuesday							
Wednesday							
Thursday							
Friday							
Saturday							
Sunday							
Total (sum of each Column)							
